# A Reflective Photonic Crystal Fiber Temperature Sensor Probe Based on Infiltration with Liquid Mixtures

**DOI:** 10.3390/s130607916

**Published:** 2013-06-20

**Authors:** Ran Wang, Jianquan Yao, Yinping Miao, Ying Lu, Degang Xu, Nannan Luan, Mayilamu Musideke, Liangcheng Duan, Congjing Hao

**Affiliations:** 1 College of Precision Instrument and Optoelectronics Engineering, Institute of Laser and Optoelectronics, Tianjin University, Tianjin 300072, China; E-Mails: wangran1986@tju.edu.cn (R.W.); jqyao@tju.edu.cn (J.Y.); luying@tju.edu.cn (Y.L.); xudegang@tju.edu.cn (D.X.); nannanluan@gmail.com (N.L.); mahira_laser@yahoo.cn (M.M.); duan072@tom.com (L.D.); cathyhcj@163.com (C.H.); 2 Key Laboratory of Optoelectronics Information Technology, Tianjin University, Ministry of Education, Tianjin 300072, China; 3 Tianjin Key Laboratory of Film Electronic and Communication Device, School of Electronics Information Engineering, Tianjin University of Technology, Tianjin 300384, China

**Keywords:** photonic crystal fiber, temperature sensor probe, liquid mixtures, finite element method, cryogenic temperature

## Abstract

In this paper, a reflective photonic crystal fiber (PCF) sensor probe for temperature measurement has been demonstrated both theoretically and experimentally. The performance of the device depends on the intensity modulation of the optical signal by liquid mixtures infiltrated into the air holes of commercial LMA-8 PCFs. The effective mode field area and the confinement loss of the probe are both proved highly temperature-dependent based on the finite element method (FEM). The experimental results show that the reflected power exhibits a linear response with a temperature sensitivity of about 1 dB/°C. The sensor probe presents a tunable temperature sensitive range due to the concentration of the mixture components. Further research illustrates that with appropriate mixtures of liquids, the probe could be developed as a cryogenic temperature sensor. The temperature sensitivity is about 0.75 dB/°C. Such a configuration is promising for a portable, low-power and all-in-fiber device for temperature or refractive index monitoring in chemical or biosensing applications.

## 1. Introduction

Recently, optical fiber sensors have become more and more attractive due to their miniaturization, electromagnetic immunity, chemically inertness, network compatibility, and the aptitude for remote or *in situ* measurements [1,2]. The appearance of photonic crystal fibers (PCFs) is a breakthrough in fiber optic technology, leading to unprecedented properties that overcome many limitations [3,4]. In contrast with traditional optical fibers, PCFs are made of single material and have several geometric parameters that can be manipulated for larger flexibility of design. With the modulation of the size and location of the cladding air holes, the characteristics of PCFs, such as mode shape, transmission spectrum, nonlinearity, dispersion and birefringence, could be tunable to manage the anticipated values [5–7]. Additionally, the existence of air holes provides the possibility to insert functional materials, the refractive index of which is dependent on external physical fields [8]. This enables further dynamic modification of the waveguide properties and provides perspectives for various all-in-fiber tunable or sensing devices [9,10].

A wide variety of fiber-optic-based temperature sensing schemes have been proposed and reported to date [11,12]. However, these traditional configurations present a number of disadvantages such as high coupling losses, costly integration, limited mechanical reliability, and difficulties in mass production. Some all-in-fiber configurations have reported to overcome the drawbacks mentioned above. Han *et al.* demonstrated an ultrasensitive PCF temperature sensor with a sensitivity of 13.1 nm/°C by introducing the avoided-crossing effect in a bent-controlled fluid-filled photonic bandgap fiber [13]. Qian *et al.* investigated a compact temperature sensor based on a fiber loop mirror combined with an alcohol-filled high-birefringence PCF. The interference spectrum of the resonant dips presented a temperature sensitivity of 6.6 nm/°C [14]. Some Fiber-Bragg-Gratings-based sensors have been reported as well. ORMOCER-coated FBGs have been investigated at cryogenic temperature (50–300 K) with a linear temperature shift of the Bragg wavelength of 2.4 pm/° C [15]. Another metal recoated FBG sensor which is insensitive to magnetic field and provides a sensitivity of about 15 pm shift/° C is demonstrated in [16]. Compared with the wavelength-modulated counterpart in [13–16], the intensity-modulated configuration only requires a laser diode source associated with an optical power meter for signal interrogation. Expensive and high precision apparatus such as the broadband amplified spontaneous emission (ASE) fiber source associated with high-resolution optical spectrum analyzers (OSAs) are not necessary. An intensity-modulated scheme with an ethanol-infiltrated PCF has been demonstrated in [17]. The sensitivity of transmitted power is experimentally determined to be 0.315 dB/°C for a 10 cm long PCF. However, as a transmission-type sensor, the optical source and the power meter are on the discrete sides of the PCF, which is not convenient in the practical measurement of ambient temperature. Both ends of the PCF need to be fusion spliced to single mode fiber, leading to higher fusion splicing loss and higher complexity of the system. Additionally, this sensor operated with a single liquid at room temperature and the detection range is not tunable for further practical applications.

In this paper, the operation of a reflective temperature sensor probe based on an intensity modulated solid core PCF has been demonstrated. The active region is a 1cm liquid mixture-infiltrated section of a commercial LMA-8 PCF. The optical source and the power meter lie on the same side of the PCF and the probe can be extended into the aimed environment under harsh conditions. The reflected power of the infiltrated PCF shows a linear response to temperature with a sensitivity of about 1 dB/°C. Additionally, with proper mixtures of temperature-sensitive liquids, this sensor probe presents a tunable detection range and could be developed for ultralow temperature measurement, the sensitivity of which is 0.75 dB/°C. The device provides useful properties such as compactness, simple design, and easy fabrication with high measurement accuracy.

## 2. Numerical Simulation and Theoretical Analysis

**Figure 1.**
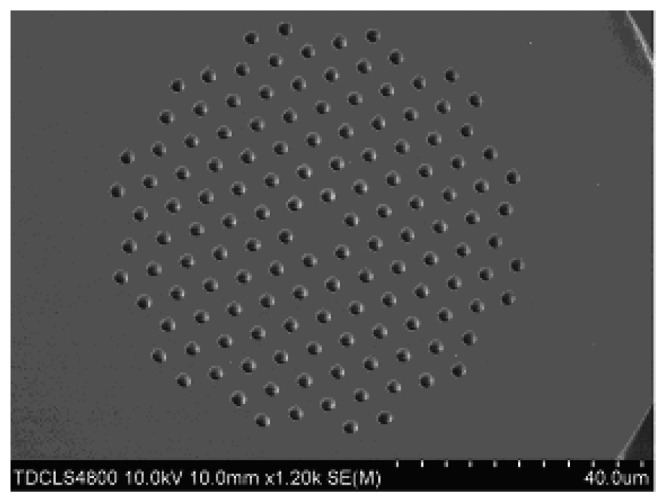
The image of LMA-8 cross section from field emission scanning electron microscopy.

The numerically simulated PCF is commercially available LMA-8 fiber [18], with a core diameter of ~8.5 μm and a lateral size of ~125 μm, surrounded by seven rings of air holes arranged in a triangular lattice. The inter-hole distance and the diameter of the air holes are Λ = 5.6 μm and D = 2.7 μm, respectively. The cross section of the LMA-8 PCF is shown in Figure 1.

Liquids such as ethanol, toluene and chloroform present high temperature-dependent optical sensitivity. PCFs infiltrated with these materials are susceptible to external temperature variations. The refractive index of the background material SiO_2_ is calculated according to the Sellmeier equation [19]. In the liquids, the temperature behavior of the refractive index is assumed as a linear approximate expression:

(1) $$n\left(\Delta T\right)=n_{0}+\frac{dn}{dT}\cdot \Delta T.$$

Here, *n_0_* denotes the refractive index given by the Sellmeier equation. Δ*T* is the difference between the absolute temperature *T* and the temperature *T_0_* at which the Sellmeier coefficients are given. The thermo-optical coefficients *dn*/*dT* amount to −5.273 × 10^−4^/°C for toluene, −6.328 × 10^−4^/°C for chloroform and −3.940 × 10^−4^/°C for ethanol [20,21]. Furthermore, they are assumed independent of the incident wavelength and temperature. Compared with the liquids above, the thermo-optical coefficient of SiO_2_ (~10^−6^/°C) is not taken into consideration. The Lorentz-Lorenz equation is used for the refractive index of the liquid mixtures [22]:

(2) $$\frac{n^{2}-1}{n^{2}+2}=\phi_{1}\frac{n_{1}^{2}-1}{n_{1}^{2}+2}+\phi_{2}\frac{n_{2}^{2}-1}{n_{2}^{2}+2}.$$

Here, *n*, *n_1_*, and *n_2_* denote the refractive index of the solution and the constituents, respectively. *ϕ*_1_ and *ϕ*_2_ are the volume fractions of the constituents and *ϕ*_2_ can be replaced by (1 − *ϕ*_1_). From the simulated modes, the effective mode area *A_eff_* is calculated by [23]:

(3) $$A_{\text{eff}}=\frac{{\left({\iint \left|E\left(x,y\right)\right|}^{2}\text{dxdy}\right)}^{2}}{{\iint \left|E\left(x,y\right)\right|}^{4}\text{dxdy}}$$

where *E*(*x*,*y*) represents the two-dimensional electric field distribution of the mode. Additionally, the confinement loss (dB/m), arising from the imaginary part of the effective complex refractive index *n_eff_*, is given as [17]:

(4) CL(dB/m)=20log10e×Im(βeff)=8.686×Im(neff)×k0.

**Figure 2.**
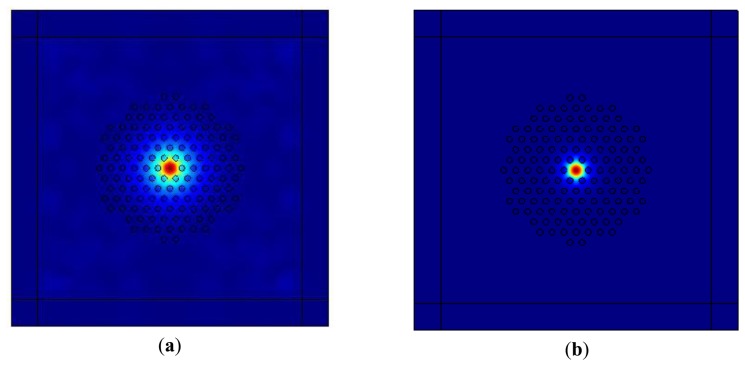
The simulated distributions of LMA-8 PCF fundamental modes with the volume ratio of chloroform and ethanol as 9:1, **(a)** at 0 °C , **(b)** at 20 °C .

Mixtures of chloroform and ethanol with a volume ratio of 9:1 have been infiltrated into the cladding air holes of LMA-8 PCFs. Light is still guided by total internal reflection (TIR) as the mixtures present lower refractive index than the fiber core. The distribution of the fundamental mode, the effective mode area *A_eff_*, and the confinement loss have been simulated based on the full-vector finite element commercial software packages COMSOL Multiphysics combined with the anisotropic Perfectly Matched Layer (PML). Figure 2 illustrates the patterns of fundamental mode in 0 °C and 20 °C . As seen from the figure, fundamental mode distribution, corresponding to the penetration of the mode field into cladding, obviously decreases with the increase of temperature, which proves that the device is sensitive to the ambient temperature.

**Figure 3.**
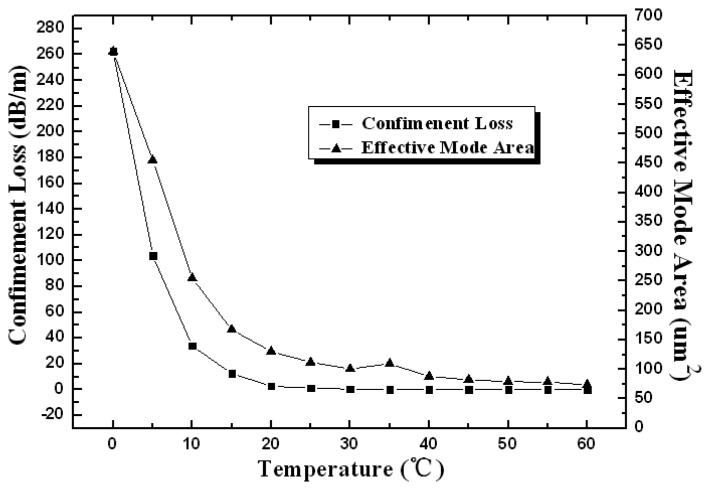
The confinement loss and effective mode area as functions of temperature.

The confinement loss and the effective mode area of the device have been illustrated in Figure 3 as functions of temperature. They are highly temperature-sensitive in the region from 0 to 20 °C. Larger effective mode area at lower temperature corresponds to more penetration of the evanescent wave into the cladding, leading to larger confinement loss of the device. As the temperature increases from 0 to 20 ° C , the refractive index of the liquid-filled cladding gradually decreases by *dn*/*dT* ~ 10^−4^/ °C according to Equation (1). The larger contrast of the refractive indices between the core and cladding results in more effective waveguide confinement, resulting in obvious decrease of the confinement loss (from 262 to 2.65 dB/m) and the effective mode area mode field (from 640 to 130 μm^2^). As the temperature rises up to 20 ° C , the fundamental mode field is well confined in the core and both parameters above present constant in the higher temperature region.

## 3. Experimental Results and Discussion

**Figure 4.**
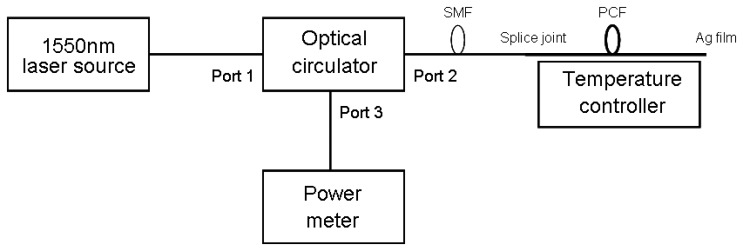
Scheme of the experimental setup for the LMA-8 PCF sensor probe.

Figure 4 shows the experimental scheme of the PCF-based sensor probe. The active region is a 1 cm commercial LMA-8 PCF liquid-infiltrated (chloroform and ethanol) simply by capillary action during several tens of minutes. The liquid-filled PCF is spliced to standard single-mode fibers (SMF) with a splicing loss of 1 dB. The other end is coated with Ag film through the common silver mirror reaction. The PCF is then placed into the temperature controller in the V-groove of an aluminum slab to avoid bending effects. The signal light from a tunable semiconductor laser source (Agilent 8164A) at the wavelength of 1,550 nm is coupled into the active region through a fiber circulator. The reflected power from the Ag film is then coupled to a digital power meter (LX Light Wave). The intensity signal is detected from 0 to 50 ° C to investigate the temperature dependence. In order to avoid the influence of the light source fluctuation, the average values from 10 repeated measurements at each temperature are used in the practical experiment.

**Figure 5.**
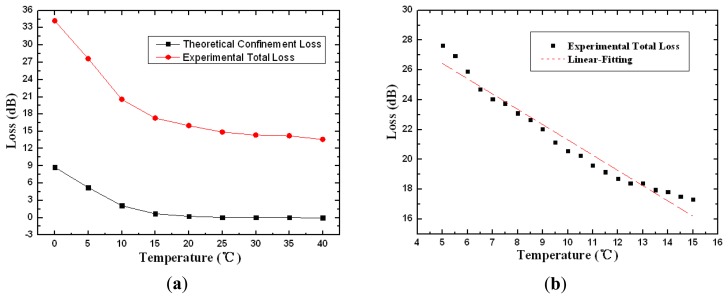
Theoretical and experimental loss **(a)** and linear fitting curves of the experimental loss **(b)** as functions of temperature with the volume ratio of chloroform and ethanol as 9:1.

Figure 5(a) shows both the theoretical confinement loss and the experimental total loss for the PCF sensor probe as a function of temperature with the volume ratio of chloroform and ethanol set at 9:1. The attenuation of the device decreases from 34.2 to 13.5 dB within the temperature transition range of 15 °C (from 0 to 15 °C ). The simulation results and the experimental measurements present a good qualitative agreement in general. It should be noted that the total loss comprises not only confinement loss but also intrinsic loss, splicing loss, and the imperfect reflection from Ag film, so it is about 15–20 dB higher than the theoretical confinement loss. The slight discrepancies arise from the instabilities of liquid fluctuation, the uneven heating of temperature controller and the influence of external environment. The linear fitting curves of the experiment total loss have been plotted in Figure 5(b) at 0.5 °C intervals. There is an approximate direct proportion relationship and the linear fitting expression is given as:

(5) $${\text{Loss}}_{1}=31.5506-1.024\;T.$$

The sensitivity corresponding to the slope is 1.024 dB/°C with the standard error 0.04686 from 5 to 15 °C . The R-squared value estimated with linear regression fits is 0.95973.

**Figure 6.**
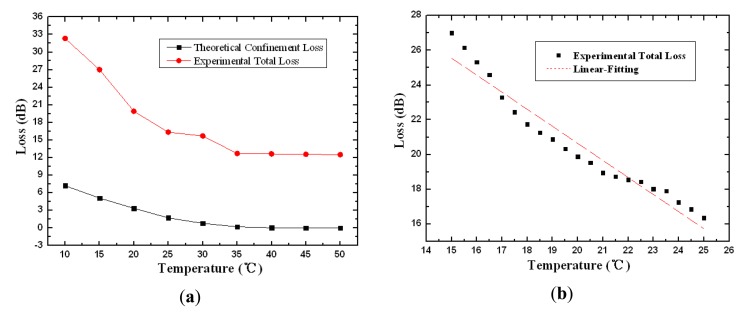
Theoretical and experimental loss **(a)** and linear fitting curves of the experimental loss **(b)** as functions of temperature with pure chloroform.

The similar simulated and experimental results have been shown in Figure 6(a), while the linear fitting curve have been plotted in Figure 6(b) with pure chloroform filled into the air holes. The attenuation decreases from 32.3 to 12.5 dB within the temperature from 10 to 25 °C . The linear fitting expression is given in the temperature sensitive range from 15 to 25 °C:

(6) $${\text{Loss}}_{2}=40.28428-0.98217\;T.$$

The sensitivity corresponding to the slope is 0.98217 dB/°C with the standard error 0.05401 and the R-squared value is 0.94281. It is worth noting that the temperature transition region in Figure 5 and Figure 6 is just the linear response range. According to Figure 3, the device presents higher sensitivity in lower temperature range. With appropriate standardization of the measurement results, it is possible to obtain the quantitative functional relationship between the loss characteristic and temperature in a broader temperature range.

The physical mechanism of the phenomenon above lies in the manipulation of the core mode by the tunable refractive index of the material infused into the air holes. In Figure 5(a) and Figure 6(a), the refractive index of the material is close to that of SiO_2_ in the low temperature region, resulting in dramatic loss and attenuation for the propagating mode. With the increase of temperature, the refractive index of liquid-filled cladding gradually decreases and is much lower than that of SiO_2_, the mode is effective confined in the fiber core and only a small percentage of the optical field penetrates into the cladding. In this case, the mode propagates through the fiber with minimal loss.

As for the tunable temperature operation adjustment, the high temperature-sensitive range corresponds to the refractive index of the cladding mixture liquids in the numerical interval from 1.430 to 1.437. For different proportions of solutions, these values could be achieved in different temperature ranges, even in extremely harsh environments for industrial applications.

**Figure 7.**
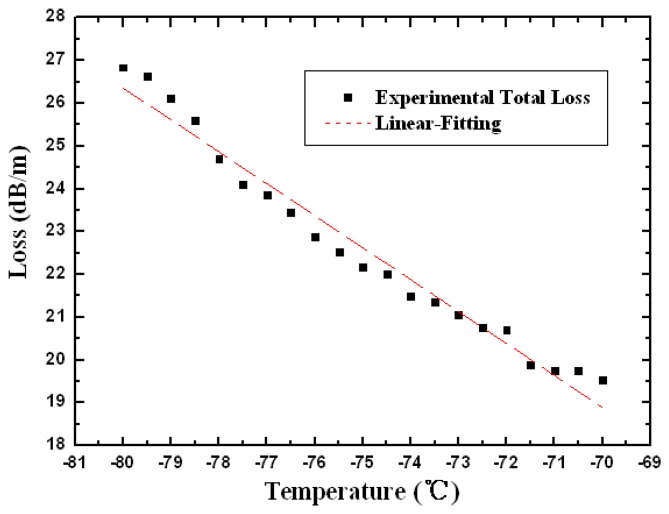
The experimental total loss and linear-fitting curve as functions of temperature in the ultralow range with a 3:7 volume ratio of toluene and ethanol.

As seen from the reference [20,21], the melting points of toluene and ethanol are −94.99 ° C and −114.1 °C , which indicate the detection limits of the device. It is believable that with an appropriate mixture of the two liquids, the sensor could be designed to operate at cryogenic temperatures. To achieve the ultralow temperature condition, the scheme of the experimental setup has been improved with liquid nitrogen for cryogenic cooling. The active region of the probe is attached to the platinum resistance thermometer sensor (STTH Pt100) for temperature monitoring. The experimental total loss and the linear fitting curve have been plotted in the Figure 7 with a 3:7 mixture of toluene and ethanol. The linear fitting expressions are given as follows:

(7) $${\text{Loss}}_{3}=-33.36671-0.7466\;T.$$

The R-squared value estimated with linear regression fits is 0.97122 and the slope of the curve, corresponding to the temperature sensitivity, is 0.7466 with the standard error 0.02871. Comparing Equation (7) with Equations (5) and (6), the sensitivity of the probe at cryogenic temperature, corresponding to the slope, is lower than that at room temperature. This is because the thermo-optical coefficient of ethanol is lower than that of toluene and chloroform according to references [20,21], and in this case, the volume fraction of ethanol is higher than in the room temperature case, reducing the temperature sensitivity of the mixture. That is the physical mechanism of the sensitivity difference between the ultralow and room temperature conditions. Such a device could be developed as an intensity-modulated thermo-optic sensor probe with a tunable temperature sensitive range, especially under harsh conditions.

In practical applications, the interrogation is carried out with commercial available optical fibers and components. The fabrication of the device is simple since it only involves cleaving and splicing. The system needs just once fusion splicing for less couple loss compared with the transmission-type scheme. Furthermore, the intensity-modulated configuration only requires a laser diode source associated with an optical power meter. The signal light passes twice through the active region due to the reflection by the silver film at the end of the PCF. This doubles the effective interaction between the light and the tunable materials, leading to higher temperature sensitivity.

## 4. Conclusions

In conclusion, the intensity-modulated all-in-fiber temperature sensor probe has been demonstrated both theoretically and experimentally. The probe, based on the thermo-optical tunability of liquid-infiltrated LMA-8 PCF, presents a linear response with a sensitivity of ˜1d B/°C. With an appropriate mixture of liquids, the sensor could be designed to operate in a desired measurable temperature range. A cryogenic temperature sensor probe has been researched experimentally as well. Such a configuration could be explored for an all-in-fiber sensor probe for temperature or refractive index monitoring with the merits of simple structure, compact configuration and easy demodulation in chemical or biosensing applications.
